# On the Use of Bootstrapped Topologies in Coalescent-Based Bayesian MCMC Inference: A Comparison of Estimation and Computational Efficiencies

**DOI:** 10.4137/ebo.s2765

**Published:** 2009-07-31

**Authors:** Allen G. Rodrigo, Peter Tsai, Helen Shearman

**Affiliations:** The Bioinformatics Institute, and The Allan Wilson Centre for Molecular Ecology and Evolution, University of Auckland, Private Bag 92019, Auckland, New Zealand. Email:a.rodrigo@auckland.ac.nz

**Keywords:** coalescent, Bayesian inference, MCMC, bootstrap, effective population size

## Abstract

Coalescent-based Bayesian Markov chain Monte Carlo (MCMC) inference generates estimates of evolutionary parameters and their posterior probability distributions. As the number of sequences increases, the length of time taken to complete an MCMC analysis increases as well. Here, we investigate an approach to distribute the MCMC analysis across a cluster of computers. To do this, we use bootstrapped topologies as fixed genealogies, perform a single MCMC analysis on each genealogy without topological rearrangements, and pool the results across all MCMC analyses. We show, through simulations, that although the standard MCMC performs better than the bootstrap-MCMC at estimating the effective population size (scaled by mutation rate), the bootstrap-MCMC returns better estimates of growth rates. Additionally, we find that our bootstrap-MCMC analyses are, on average, 37 times faster for equivalent effective sample sizes.

## Introduction

The coalescent is a mathematical description of the genealogy of a sample of sequences from a Wright-Fisher population. Kingman^1^,^2^ showed that the times to common ancestry of any pair of lineages, measured from present to past, can be approximated by exponential random variables with the expected time proportional to 2*N*/*i*(*i* − 1), where *N* is the effective size of the population, and *i* is the number of lineages that have yet to coalesce as we move from the tips to the root of the tree. If the population is subdivided and/or has changed in size, then these intervals are functions of migration rates and/or growth rates, respectively. As a means of inferring population genetic parameters, its use has grown, and this growth has been spurred by our increasing ability to sample sequences from many individuals in a population. We can derive a maximum-likelihood estimate (MLE) of the effective population size (scaled by the mutation rate) by finding the value that maximises the probability of observing the series of coalescent intervals obtained with our sample genealogy, *G*, given by

(1) $$P(G|\Theta )=\frac{2^{n-1}}{\Theta^{n-1}}\prod_{i=2}^{n}\text{exp}\left(-\frac{k_{i}(k_{i}-1)}{\Theta }\rho_{i}\right)dt$$

where time, *t*, is measured in substitutions, *ρ_i_* is the length of the *i*th branch, also in substitutions, and Θ ∞ 2*N μ* (*μ* is the mutation rate, and the proportionality constant depends on whether the population is haploid or diploid).

Of course, the genealogy is seldom known with certainty, and the approach adopted over the last few years has been to develop clever computational methods that integrate over all genealogies, weighting each genealogy by its likelihood:^3^,^4^

(2) $$P(D|\Theta )=\int_{G}P(D|G)P(G|\Theta )dG$$

The term *P*(*D* | *G*) is the standard phylogenetic likelihood.

This approach also applies to the Bayesian methods that have been developed.^5^ Here, the aim is to recover the posterior probability distribution, *P*(Θ | *D*) ∞ *P*(*D* | Θ)*P*(Θ), where *P*(Θ) is the prior distribution of Θ. Bayesian methods that have been developed to take account of the uncertainty in the genealogy rely on Markov chain Monte Carlo (MCMC) integration with or without importance sampling. With MCMC, a genealogy or a parameter value, *X*′*_i_*, is perturbed according to some proposal distribution or strategy to *X*′*_i_*_+1_, the posterior probability of *X*′*_i_*_+1_ is calculated, and *X*′*_i_*_+1_ is accepted or rejected based on the ratio of the posterior probabilities of *X*′*_i_*_+1_ and *X*′*_i_* and the proposal probabilities of moving from *X*′*_i_* to *X*′*_i_*_+1_ and *X*′*_i_*_+1_ to *X*′*_i_*.

MCMC is a powerful computational technique that is naturally suited to Bayesian inference because, in its simplest and most intuitive form, it delivers a probability distribution of parameter values instead of one value that maximizes some function. In this paper, we will focus on MCMC and its use in coalescent-based Bayesian inference. There are many issues relating to the performance of MCMC: how do we know when the Markov chain has converged to the target distribution, how frequently should we sample, how long should chains be, and so on. We will ignore all of these, largely because there are many good texts, primers and reviews on these topics. Instead, we will focus on a method that permits us to distribute our MCMC coalescent integration across a computational cluster to achieve an increase in the speed of execution.

There are three main reasons to use cluster-based computing for MCMC: to assist with mixing, to increase the speed of the MCMC procedure, and as a check for convergence. For instance, MrBayes^6^ uses a computational cluster to perform multi-chain Metropolis Coupled MCMC, permitting samples to mix across different chains. BayesPhylogenies^7^ uses a computational cluster to calculate the likelihoods of parts of the data, thus increasing the speed of execution. Finally, it is also possible to run several MCMC chains of the same data on a cluster to check for convergence to the same target distribution.

In this paper, we examine an approach first proposed by Felsenstein^8^ which involves the use of bootstrap trees. This method has not been implemented in any existing software, nor has it been tested to any great extent. Our aim is to study the properties of estimates derived using this approach, in an attempt to determine whether the relative benefits of increased computational speed outweigh any loss in estimation efficiency.

## The procedure

We begin by noting that a genealogy, *G*, can be characterized by an ordered history, *H*, that denotes the order in which labeled lineages coalesce, and a vector, *C*, of coalescent intervals. We write *G* = {*H*, *C*}, and

(3) $$P(D|\Theta )=\sum_{H}\int_{C=0}^{\infty }P(D|\{H,C\})P(\{H,C\}|\Theta )dC$$

Clearly, the likelihood of Θ will be influenced by ordered histories that are, themselves, most likely— the leftmost term within the integral indicates this. One way to recover the set of “likely” ordered histories is to use the histories of bootstrapped trees. By bootstrapping the data as proposed by Felsenstein^9^ and reconstructing the sequence phylogenies to obtain the set, *B*, of bootstrapped histories, we can write:

(4) $$P(D|\Theta )\approx \sum_{B}\int_{C=0}^{\infty }P(D|\{B,C\})P(\{B,C\}|\Theta )dC$$

As before, to obtain the posterior probability of Θ, we have

(5) $$\begin{array}{l} P(\Theta |D)\propto P(D|\Theta )P(\Theta )P(\Theta |D)\\ \approx \sum_{B}Z\int_{C=0}^{\infty }P(D|\{B,C\})P(\{B,C\}|\Theta )P(\Theta )dC \end{array}$$

where *Z* is an unknown normalizing constant that cancels out in the MCMC process. Eqn 5 immediately suggests a strategy: distribute to each node on a computational cluster a fixed history from *B*, turn off topological rearrangements, and pool the posterior distributions obtained from all nodes. When topological rearrangements are turned off, the topology of the genealogy remains fixed for the entire MCMC run. The value of this approach is two-fold: (1) it allows a parallel implementation of MCMC; and (2) for each bootstrapped history, MCMC perturbations focus only on continuous parameters (i.e. branch lengths, coalescent parameters, and substitution model parameters). In our simulations, this delivers an increase in computational speed.

Of course, as Felsenstein^8^ noted, there is no guarantee that the bootstrap histories will be the “more likely” histories in any technical sense, but intuition suggests that they will constitute an assemblage of trees with reasonably high likelihoods. As an aside, it is worth noting that Kuhner, Yamato, and Felsenstein^4^ argued against this approach because bootstrap trees admit zero-length branches and estimates of Θ based on these branches will be indeterminate under the coalescent likelihood (Eqn. 1). However, what we have done here is allow the MCMC procedure to alter the branch lengths, so we effectively strip the branch-lengths away leaving only the history.

## Simulations

Seventy haploid sequences, each 1000 bases long, were generated randomly under the coalescent process using SimCoal 2.^10^ The constant population size was set at 100,000. The mutation rate was 1.5 × 10^−6^ mutations per site per generation. Ten replicates were generated. This process was also repeated using sample sizes of 140 and 210 sequences. Sequences were also generated assuming an exponentially growing population with a current (or terminal) population size of 100,000 increasing at a rate of 0.0005, again with ten replicates for samples of 70, 140, and 210 sequences.

For each data set, 100 bootstrap trees were generated using PHYML v1.2.2.^11^ A BIONJ distance-based tree is used as the starting tree in PHYML and optimized under a HKY substitution model using maximum likelihood with four substitution rate categories. All the other parameters (e.g. transition/transversion ratio, proportion of invariable sites and gamma distribution shape-parameter) were estimated using PHYML.

Bootstrapped trees were midpoint-rooted and were then analyzed using BEAST^12^ with shortened chain length (3 million). Thus, we performed 100 MCMC runs for each data set and the topology used in each run was fixed on a different bootstrap tree topology. MCMC samples from all runs on the set of bootstrapped topologies were then combined to obtain the final marginal distributions. Additionally, each original (non-bootstrapped) dataset was analysed with BEAST allowing topological rearrangements, as a comparison. The number of generations for these “standard” MCMC analyses were set to allow the Effective Sample Size (ESS) to approximate that obtained using the bootstrap- MCMC analyses. Generally, MCMC chains for the standard analyses ran for 60–420 million generations. For all analyses, parameters of the substitution model were allowed to vary, uniform priors were used for all continuous parameter variables, the chains were sampled every 5000 generations, and the first 10% were discarded as burn-in values. All MCMC analyses were run on a 10-node SGI Altix XE320 cluster, with each node consisting of 2 × Quad Core Xeon 2.8 GHz processors. In total, 80 cores were available.

Analyses of median estimates of Θ and growth rates, *G* (where applicable) were performed using JMP 7.0.^13^ To analyse the simulations, mixed-model nested Analyses of Variance (ANOVAs) were used, with Method (either Standard MCMC or Bootstrap MCMC) and Simulation (70 sequences, 140 sequences or 210 sequences) as fixed categorical effects, and replicate as a random effect nested within Simulation. The interaction effect between Method and Simulation was also a factor in the model.

## Results

Results for all simulations are given in Tables 1 and 2. For constant sized populations, the bootstrap-MCMC estimates of Θ averaged 0.163, compared to the true value of 0.150—this equates to about a 10% difference between the true and estimated values. In contrast, the standard MCMC returned an average of 0.149, a difference of less than 1%. The difference in estimation between the bootstrap-MCMC and the standard MCMC was statistically significant (*p*-value < 0.001). ANOVA indicated there was no significant difference as sequence numbers changed, nor was there interaction between Method (i.e. bootstrap-MCMC vs standard MCMC) and Simulation (i.e. numbers of sequences). Also, 4 of the 30 95%HPDs obtained using the bootstrap-MCMC did not enclose the true value, whereas only 1 of the 30 95%HPDs of the standard MCMC excluded the true value, although this is not statistically significant at the 5% level.

In contrast, when we compare the bootstrap- MCMC and the standard MCMC estimates of growth rate, we find that there is a statistically significant interaction effect between Method and Simulation (*p*-value < 0.01), with the standard MCMC performing more poorly as numbers of sequences increased. Also, the 95%HPDs of bootstrap-MCMC analyses enclose the true value of growth rate more frequently (23/30) than those obtained with the standard MCMC (15/30; *p*-value < 0.05). The bias seen in the standard MCMC is not surprising: Kuhner et al^14^ demonstrated that the ML estimates of growth rate tend to be significantly biased upwards. We expect Bayesian estimates to show the same tendency, particularly with uninformative priors.

Whereas the standard MCMC does not appear to estimate growth rates as well as the bootstrap-MCMC, it seems to estimate the terminal value of Θ better than the bootstrap-MCMC, and theses estimates improve as more sequences are added. Of the 30 95% HPDs, 7 of the bootstrap-MCMC HPDs exclude the true value, whereas all standard MCMC HPDs enclose the true value (*p*-value < 0.01).

**Figure 1:**
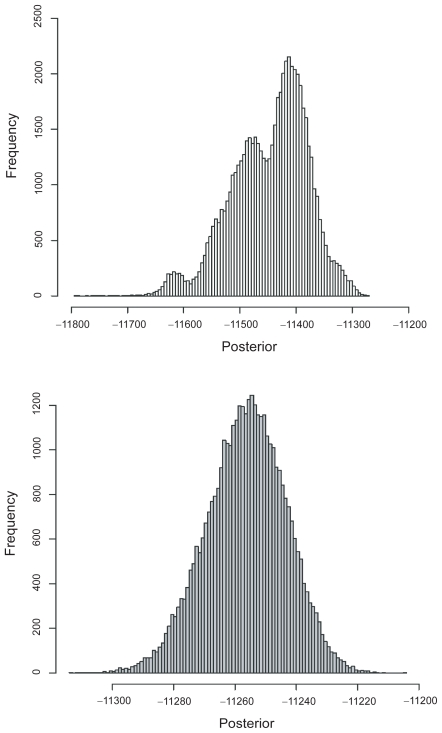
Posterior distribution from bootstrap-MCMC and standard-MCMC. Example of the log-posterior probability distribution from both bootstrap- MCMC (top) and standard-MCMC (below) obtained with 210 sequences simulated with a constant population size. Note also the difference in scales of the horizontal axes.

Interestingly, the frequency distribution of posterior probabilities is multimodal for the bootstrap-MCMC and unimodal for the standard MCMC (Figs. 1A, B). In retrospect, this is not surprising, since only a small part of topology space is explored under the bootstrap-MCMC. It is worth noting, however, that the number of modes on the marginal distribution of log-posterior probabilities obtained using the bootstrap-MCMC does not correspond to the number of unique topologies obtained using the bootstrap. There are more topologies obtained than modes on the marginal distribution of posterior probabilities. Also, it is worth pointing out that the bootstrap- MCMC obtains lower log-posterior probabilities than the standard MCMC.

Finally, if we compare the times of the runs, we find that if the MCMC run for 100 bootstrapped topologies was performed on a 80-core cluster, the bootstrap MCMC took an average of just over an hour (61 mins, range: 44–94 mins) to obtain an average ESS of 17372; in contrast, the standard MCMC took, on average, 37 hrs (2216 mins, range: 1446–4373 mins) to obtain approximately the same ESS (17888).

## Discussion

In this paper, we explore the properties of an approach to coalescent-based Bayesian MCMC estimation of evolutionary parameters that begins with a set of bootstrapped topologies which remains fixed throughout the analyses. Distributing these topologies across a cluster of computers affords up to a 37-fold increase in computational speed. In terms of estimation efficiency, the results are mixed: whereas the standard MCMC performs better at estimating Θ, it fails to estimate growth rate as well as the bootstrap-MCMC.

It is fair to say that in the absence of any analytic solution, most estimation methods in phylogenetics and evolutionary genetics rely on heuristic procedures. MCMC itself is a heuristic procedure that only guarantees convergence to the target distribution (generally, the posterior probabilities), under appropriate conditions, without any specification of when that convergence will be reached. Consequently, we never know that we are sampling from the correct distribution without running additional tests. Heuristic methods are useful because, typically, a researcher is prepared to make a trade-off between the time it takes to run an analysis (i.e. computational efficiency) and the degree of uncertainty in the estimates (i.e. estimation efficiency). This is particularly true as we accumulate more sequences, because standard MCMC analyses will require longer times to run. As noted above, the method described here achieves a phenomenal speed increase with our simulations.

The method proposed here can almost certainly be improved. If, instead of using bootstrapped trees, we use trees that are most likely, or nearly most-likely, then we will get closer to essence of the procedure described above. After all, we only use bootstrap trees because we think that these are going to be in the neighborhood of the likelihood peak. Also, if instead of midpoint rooting our bootstrap trees, we found the root that was the most likely under some clock-constraint, we would again have better topologies to work with. However, in both these instances, we would take time to obtain our set of topologies, and this in turn would defeat the purpose of the exercise: the rapid delivery of estimates of evolutionary parameters with reasonable coverage properties. One possible solution, suggested by a reviewer, is to use UPGMA to build the starting topologies. The value of UPGMA is that the root for the tree is found naturally as part of the agglomerative process. UPGMA works well when a strict molecular clock applies (as in our simulations), but performs badly when there is lineage-specific rate variation. We repeated our analyses using UPGMA, but found no substantial differences to the patterns obtained with mid-point rooting, except that for growth rates, the bootstrap MCMC performed more poorly than the standard MCMC (data not shown).

Of course, the gains in computational efficiency of the method described here depend on access to a computational cluster. Such availability is no longer an issue in most research institutions. There are a variety of strategies that can be used to distribute MCMC analyses across a computational cluster. The simulated annealing literature also has distributed computing approaches that warrant exploration.^15^ In fact, the simplest approach may be to run multiple independent chains, and pool the posterior distributions, but there are two problems with this strategy: (a) each chain needs to burn in, and (b) there is no sharing of information across chains. Other strategies attempt to correct for these shortcomings, but arguably, a synthesis of several methods may be needed to deliver a significant speed increase. For instance, before the chain has converged, Metropolis Coupled MCMC may be appropriate, but after the burn-in period, pooling the distributions from several different and independent chains can be used to increase the effective sample size. Recently, the paper by Lakner et al^16^ examined the mixing and convergence characteristics of different MCMC topological rearrangements. They concluded that mixing and burn-in may be improved by a hybrid approach with different moves applied at different parts of the chain. Most recently, Suchard and Rambaut^17^ have demonstrated a significant speed increase with BEAST by deploying parts of the analysis on Graphics Processing Units (GPUs). Interest in GPU computing is increasing rapidly, and there is the potential for significant speed gains; the drawback is that parallelization has to be implemented in a particular way because of the constraints of GPU architecture. Alternatively, if we are willing to obtain good but “approximate” posterior distributions, then bootstrapping as we have applied it here, may be the answer.

## Figures and Tables

**Table 1:** Parameter estimated from sequences under constant growth rate using both bootstrap-MCMC and standard-MCMC. The true value of **Θ** is *Nμ* = 0.15.

Simulation	Est. θ boot.— Mean (Median)	Est. θ Full— Mean (Median)	θ 95% HPD—Bootstrap (Standard)	Post. ESS—Bootstrap (Standard)
70 Sequences 0	0.171 (0.169)	0.167 (0.165)	0.130, 0.217 (0.126, 0.210)	17650 (22910)
70 Sequences 1	0.157 (0.155)	0.143 (0.142)	0.118, 0.199 (0.108, 0.181)	29820 (32180)
70 Sequences 2	0.166 (0.164)	0.150 (0.148)	0.126, 0.210 (0.114, 0.189)	26390 (23610)
70 Sequences 3	0.175 (0.173)	0.141 (0.140)	0.125, 0.228 (0.108, 0.179)	13760 (11630)
70 Sequences 4	0.196 (0.194)	0.175 (0.173)	0.148, 0.248 (0.131, 0.218)	3002 (3648)
70 Sequences 5	0.169 (0.166)	0.146 (0.145)	0.123, 0.220 (0.111, 0.185)	25470 (25110)
70 Sequences 6	0.155 (0.153)	0.154 (0.152)	0.117, 0.194 (0.117, 0.194)	42420 (36840)
70 Sequences 7	0.125 (0.124)	0.124 (0.122)	0.095, 0.159 (0.093, 0.156)	31040 (33920)
70 Sequences 8	0.130 (0.128)	0.128 (0.126)	0.098, 0.164 (0.097, 0.162)	40670 (38470)
70 Sequences 9	0.158 (0.156)	0.149 (0.147)	0.117, 0.199 (0.111, 0.186)	35480 (34940)
140 Sequences 0	0.153 (0.152)	0.147 (0.146)	0.125, 0.182 (0.120, 0.175)	25550 (27850)
140 Sequences 1	0.141 (0.140)	0.119 (0.118)	0.112, 0.172 (0.097, 0.142)	12230 (13000)
140 Sequences 2	0.151 (0.150)	0.145 (0.145)	0.124, 0.181 (0.119, 0.172)	26640 (22830)
140 Sequences 3	0.191 (0.189)	0.169 (0.168)	0.154, 0.228 (0.139, 0.201)	17660 (18690)
140 Sequences 4	0.158 (0.157)	0.153 (0.152)	0.129, 0.189 (0.125, 0.182)	26390 (27460)
140 Sequences 5	0.133 (0.132)	0.128 (0.127)	0.108, 0.160 (0.105, 0.153)	22860 (19510)
140 Sequences 6	0.171 (0.170)	0.135 (0.135)	0.134, 0.209 (0.112, 0.162)	8927 (9467)
140 Sequences 7	0.180 (0.178)	0.159 (0.158)	0.146, 0.217 (0.129, 0.189)	16210 (16420)
140 Sequences 8	0.187 (0.185)	0.174 (0.173)	0.151, 0.225 (0.144, 0.207)	10200 (10780)
140 Sequences 9	0.172 (0.171)	0.152 (0.151)	0.140, 0.208 (0.123, 0.180)	10240 (11550)
210 Sequences 0	0.176 (0.175)	0.150 (0.150)	0.147, 0.206 (0.128, 0.175)	4032 (3953)
210 Sequences 1	0.159 (0.158)	0.147 (0.146)	0.134, 0.185 (0.124, 0.170)	14850 (23130)
210 Sequences 2	0.174 (0.172)	0.147 (0.147)	0.141, 0.211 (0.125, 0.171)	8089 (8350)
210 Sequences 3	0.159 (0.158)	0.150 (0.149)	0.134, 0.185 (0.127, 0.174)	16700 (25630)
210 Sequences 4	0.186 (0.185)	0.174 (0.173)	0.156, 0.215 (0.146, 0.200)	3325 (3984)
210 Sequences 5	0.160 (0.158)	0.142 (0.141)	0.129, 0.196 (0.121, 0.165)	14150 (14650)
210 Sequences 6	0.168 (0.167)	0.159 (0.159)	0.142, 0.195 (0.134, 0.183)	16160 (15320)
210 Sequences 7	0.166 (0.165)	0.158 (0.158)	0.140, 0.193 (0.134, 0.185)	17630 (18850)
210 Sequences 8	0.180 (0.179)	0.163 (0.162)	0.152, 0.209 (0.139, 0.188)	15220 (18880)
210 Sequences 9	0.160 (0.159)	0.151 (0.151)	0.135, 0.186 (0.127, 0.174)	17120 (16750)

**Table 2:** Parameters estimated from sequences under exponential growth rate using both bootstrap-MCMC and standard-MCMC. The true value of **Θ** is *Nμ* = 0.15, and the true value of G is *Ng* = 500.

Simulation	Est. θ boot.— Mean (Median)	Est. θ Full— Mean (Median)	Est. G. boot— Mean (Median)	Est. G. Full— Mean (Median)	θ 95% HPD—Bootstrap (Standard)	G. 95% HPD— Bootstrap (Standard)	Post. ESS—Bootstrap (Standard)
70 GSequences 0	0.181 (0.168)	0.132 (0.126)	434.679 (425.746)	370.959 (366.815)	0.085, 0.311 (0.071, 0.201)	251.860, 631.251 (221.913, 520.012)	16794 (12050)
70 GSequences 1	0.254 (0.240)	0.202 (0.193)	434.812 (430.352)	397.602 (394.314)	0.121, 0.411 (0.107, 0.311)	285.788, 597.187 (262.828, 534.213)	14380 (11520)
70 GSequences 2	0.238 (0.224)	0.201 (0.191)	484.164 (478.187)	465.142 (461.059)	0.113, 0.391 (0.103, 0.320)	319.461, 667.636 (301.304, 632.454)	27940 (32550)
70 GSequences 3	0.174 (0.165)	0.141 (0.135)	380.629 (376.169)	352.246 (348.381)	0.086, 0.282 (0.077, 0.215)	232.665, 540.039 (216.199, 494.499)	30690 (37260)
70 GSequences 4	0.279 (0.262)	0.234 (0.222)	494.104 (489.128)	471.440 (467.070)	0.132, 0.468 (0.118, 0.376)	329.325, 672.809 (311.627, 636.543)	24700 (18650)
70 GSequences 5	0.236 (0.222)	0.189 (0.180)	499.410 (493.513)	465.967 (460.956)	0.114, 0.393 (0.098, 0.303)	323.513, 690.004 (305.06, 641.080)	26370 (24340)
70 GSequences 6	0.192 (0.183)	0.179 (0.171)	422.840 (418.716)	429.507 (425.558)	0.092, 0.303 (0.094, 0.278)	270.336, 582.810 (284.913, 591.731)	12920 (11840)
70 GSequences 7	0.214 (0.201)	0.176 (0.168)	438.542 (432.191)	406.301 (401.038)	0.101, 0.359 (0.093, 0.276)	263.157, 616.865 (250.038, 566.817)	16480 (12074)
70 GSequences 8	0.336 (0.307)	0.236 (0.223)	585.284 (577.157)	512.506 (506.613)	0.133, 0.606 (0.113, 0.385)	371.421, 809.052 (330.425, 692.880)	10490 (9643)
70 GSequences 9	0.252 (0.239)	0.207 (0.199)	416.132 (412.133)	392.367 (388.522)	0.126, 0.404 (0.109, 0.317)	273.342, 561.178 (262.454, 527.924)	13290 (10250)
140 GSequences 0	0.227 (0.220)	0.161 (0.158)	433.741 (429.693)	360.669 (358.054)	0.142, 0.327 (0.112, 0.217)	294.335, 576.537 (250.416, 479.588)	6995 (6744)
140 GSequences 1	0.228 (0.222)	0.160 (0.158)	436.595 (432.981)	361.241 (358.454)	0.246, 0.326 (0.109, 0.217)	299.991, 577.365 (248.455, 480.345)	7989 (8188)
140 GSequences 2	0.240 (0.233)	0.167 (0.164)	473.610 (469.566)	384.907 (382.058)	0.148, 0.346 (0.116, 0.229)	330.771, 622.735 (270.311, 507.224)	6509 (6968)
140 GSequences 3	0.254 (0.248)	0.200 (0.196)	448.674 (445.572)	409.473 (406.482)	0.162, 0.360 (0.135, 0.273)	321.012, 582.177 (290.712, 533.648)	13600 (15300)
140 GSequences 4	0.199 (0.194)	0.157 (0.154)	365.991 (363.410)	323.510 (321.658)	0.131, 0.276 (0.109, 0.210)	254.410, 483.394 (221.039, 426.570)	14790 (16150)
140 GSequences 5	0.192 (0.188)	0.155 (0.152)	318.666 (316.574)	285.561 (283.529)	0.130, 0.262 (0.109, 0.204)	221.148, 419.749 (195.243, 376.003)	7522 (7017)
140 GSequences 6	0.160 (0.157)	0.127 (0.125)	354.315 (350.023)	318.985 (316.645)	0.106, 0.223 (0.085, 0.171)	230.170, 480.825 (212.019, 431.861)	9345 (13690)
140 GSequences 7	0.218 (0.212)	0.166 (0.163)	367.546 (365.015)	323.169 (321.146)	0.137, 0.306 (0.116, 0.222)	251.669, 493.619 (226.859, 422.724)	7939 (10050)
140 GSequences 8	0.265 (0.257)	0.197 (0.193)	421.904 (418.548)	366.210 (363.716)	0.163, 0.379 (0.132, 0.268)	293.196, 549.427 (259.492, 480.724)	13890 (15680)
140 GSequences 9	0.245 (0.239)	0.179 (0.175)	418.412 (415.02)	347.147 (344.787)	0.155, 0.348 (0.122, 0.241)	292.941, 547.342 (239.858, 454.721)	12920 (14450)
210 GSequences 0	0.256 (0.251)	0.182 (0.180)	482.733 (479.288)	407.266 (404.506)	0.173, 0.346 (0.135, 0.237)	354.561, 622.448 (294.541, 523.521)	5947 (7109)
210 GSequences 1	0.225 (0.219)	0.154 (0.153)	387.966 (384.962)	314.526 (313.176)	0.149, 0.308 (0.115, 0.196)	259.056, 526.567 (223.560, 412.111)	3683 (3272)
210 GSequences 2	0.199 (0.195)	0.150 (0.148)	336.966 (334.123)	289.065 (287.505)	0.142, 0.264 (0.114, 0.190)	231.906, 440.254 (205.937, 367.878)	6124 (6282)
210 GSequences 3	0.155 (0.153)	0.124 (0.123)	293.805 (291.854)	262.915 (261.456)	0.113, 0.200 (0.094, 0.156)	291.854, 390.218 (179.046, 350.422)	11250 (11110)
210 GSequences 4	0.207 (0.202)	0.140 (0.138)	465.902 (462.193)	367.540 (365.828)	0.139, 0.285 (0.103, 0.179)	323.570, 616.338 (256.036, 481.543)	8031 (7640)
210 GSequences 5	0.239 (0.232)	0.166 (0.163)	544.848 (540.033)	476.366 (473.416)	0.149, 0.341 (0.120, 0.216)	375.516, 724.607 (342.172, 610.708)	3740 (4139)
210 GSequences 6	0.273 (0.267)	0.207 (0.205)	473.015 (470.211)	424.283 (422.115)	0.187, 0.368 (0.150, 0.268)	345.418, 602.284 (313.863, 540.195)	9274 (7088)
210 GSequences 7	0.294 (0.287)	0.200 (0.187)	530.482 (527.17)	442.856 (440.409)	0.195, 0.407 (0.144, 0.259)	384.004, 682.460 (322.246, 565.085)	7618 (7488)
210 GSequences 8	0.205 (0.200)	0.151 (0.150)	390.942 (386.986)	333.692 (332.128)	0.138, 0.280 (0.113, 0.193)	267.469, 525.530 (235.079, 434.877)	6268 (5803)
210 GSequences 9	0.199 (0.195)	0.144 (0.142)	364.931 (362.487)	294.125 (292.573)	0.139, 0.264 (0.106, 0.180)	255.888, 475.197 (204.590, 385.250)	7895 (8876)
